# Evaluating Brief Behavioural Activation for depression in adolescents with acquired brain injury using a single‐case experimental design

**DOI:** 10.1002/jcv2.70030

**Published:** 2025-08-13

**Authors:** Conor R. O’Brien, Jenny Limond, Shirley Reynolds, Laura Pass, Anna‐Lynne R. Adlam

**Affiliations:** ^1^ Psychology University of Exeter Exeter UK; ^2^ School of Psychology and Clinical Language Sciences University of Reading Reading UK; ^3^ Department of Clinical Psychology and Psychological Therapies University of East Anglia Norwich UK

**Keywords:** acquired brain injury, adolescents, Brief Behavioural Activation, depression

## Abstract

**Background:**

Adolescents who have had an acquired brain injury (ABI) commonly experience depression. Brief Behavioural Activation (Brief BA) is a successful, values‐based intervention for managing depression in neurotypical adolescents. This study investigated the effectiveness of Brief BA, using a single‐case experimental design, with adolescents experiencing depression following ABI.

**Methods:**

Five adolescents, one male and four female, aged 14–17 years and with ABI, completed a 6‐week course of Brief BA. The primary outcome measures were mean daily activity scores out of 10 for ‘achievement’, 'closeness' and ‘enjoyment’ (mean achievement, closeness and enjoyment scores; MACES). MACES were collected daily for 9 weeks, comprising at least 2 weeks at baseline and at least 6 weeks during the intervention. Secondary outcome measures of depression, quality of life (QoL), and participation were collected once at baseline, immediately post‐treatment, and at a 4‐week follow‐up.

**Results:**

Two participants showed a significant increase in enjoyment scores and one participant showed a significant increase in closeness scores. No other significant differences were noted for MACES. All participants reported significant reliable improvement in depression scores at their follow‐up sessions, with three showing clinically significant improvement. Three participants reported reliable improvement in QoL. All parents reported reliable improvement in participants' depression and QoL scores. No significant changes were noted for participation scores.

**Conclusion:**

The significant changes in closeness and enjoyment scores following intervention suggest Brief BA may encourage positive behavioural change for adolescents with depression following ABI. Discussions explore the potential role of insight through linking valued activities with mood and positive reinforcement, leading to an improvement on depression and QoL outcomes. Charities and services providing low‐intensity interventions might want to consider trialling Brief BA for this population. Future research suggestions, such as investigating Brief BA for depression linked with more diverse neurological conditions, are discussed.

## Key Points

- Adolescents with acquired brain injury (ABI) commonly experience difficulties with depression due to neurological and psychosocial changes; most notably fatigue, poor goal attainment, poor self‐regulation and low participation.
- Brief Behavioural Activation (Brief BA) is a successful behavioural treatment for neurotypical adolescents with depression, targeting valued activities to increase ‘achievement’, ‘closeness’ and ‘enjoyment’.
- For adolescents with ABI, the current study showed that Brief BA improved depression symptoms for all participants, the quality of life for most participants, and was largely acceptable.
- There is evidence to suggest that targeting ‘achievement’, ‘closeness’ and ‘enjoyment’ may have facilitated improvement.
- The main clinical implication is that adolescents with ABI could access mainstream behavioural interventions like Brief BA with minor adjustments.

## INTRODUCTION

Children and young people (CYP) who have had an acquired brain injury (ABI) commonly experience depression, with reported prevalence rates of 20%–25% (Hendry et al., 2020; Schachar et al., 2015). The cognitive and behavioural impact of the ABI, psychological adjustment difficulties, and a reduced quality of life (QoL) compared with peers make CYP with ABI more at risk of developing depression than neurotypical adolescents (Connell et al., 2018).

Following injury, neurological changes, such as damage to neuronal pathways and lesions, are linked with apathy, emotional dysregulation, and difficulty initiating activities (Fayed et al., 2019). Damage to the hypothalamic‐pituitary‐adrenal axis is a common complication following ABI; the neurological changes and likelihood of cascade effects, such as lower stress tolerance, can often result in depression (Tapp et al., 2019). Psychosocial changes, such as adjustment to ABI sequelae, are also linked to an increased likelihood of depression in ABI survivors (Farner et al., 2010). Other factors that potentially contribute to depression for CYP following an ABI are lower social participation, fatigue, poorer goal attainment and self‐regulation; all of which are common in CYP with ABI (Bedell & Dumas, 2004; Cantor et al., 2008; Hart & Evans, 2006).

Psychological support is a key part of suggested rehabilitation models, particularly for CYP with ABI (Limond et al., 2014). The effectiveness of psychological interventions, such as cognitive behavioural therapy (CBT), has been explored for treating depression in adults with ABI, with positive outcomes (Stalder‐Lüthy et al., 2013). In a recent meta‐analysis, CBT‐based interventions adapted for CYP have been cited as effective for treating depression in adolescents with traumatic brain injury across five reported studies (Gómez‐de‐Regil et al., 2019) but there is limited research on psychological therapies in CYP with ABI of non‐traumatic aetiologies. Furthermore, National Health Service (NHS) mental health resources in the UK are already very stretched (Gilburt, 2018). Whilst primary care services have been and are improving rates of ‘access’ to therapies for depression over the last few years (NHS England, 2019), CYP with ABI are often automatically excluded from services providing psychological and behavioural therapies.

Brief Behavioural Activation (Brief BA) is a clinically suitable, efficient, readily available and acceptable form of BA for neurotypical adolescents, with excellent rates of adherence and good outcomes for patients (Pass et al., 2018). Brief BA mainly focuses on identifying behavioural patterns linked with depression symptoms and modifying or replacing them to include activities that give the recipient an increased sense of ‘achievement’, ‘closeness’ and/or ‘enjoyment’, depending on their goals and values. ‘Achievement’ can be defined as a feeling of success when completing an activity, ‘closeness’ as a feeling of connection with someone or something (e.g., religion, spirituality, etc.), and ‘enjoyment’ as a feeling of having fun (Reynolds & Pass, 2021).

There is no available research into the efficacy of any type of BA for depression in CYP with ABI. However, recent research into BA for depression in adults with ABI has shown promising results (Gertler & Tate, 2019). In a non‐ABI population, BA is comparable in effectiveness to CBT for treating depression in adults (Ekers et al., 2008; Richards et al., 2016). As for adolescents with depression, Weisz et al. (2006) have suggested that psychotherapies with cognitive components are no more effective than those without. This was supported by a study comparing BA to ‘evidence‐based’ therapies for adolescent depression; CBT and interpersonal therapy (McCauley et al., 2016). The relative ‘simplicity’ of BA compared with more cognitive approaches is considered to make it more amenable to adolescents than more cognitive approaches, particularly as an adolescent's cognitive function is continually developing (Pass et al., 2015).

CYP with ABI typically exhibit lower activity levels than their peers (van Markus‐Doornbosch et al., 2019), mainly due to poor motivation, anhedonia, lack of initiation, and social withdrawal (Ownsworth & Oei, 2009). CYP with ABI also have difficulty with planning, initiating activities, and self‐regulation (Middleton, 2001), which can affect participation (Cook et al., 2008). Parental anxiety about the risks of returning to normal activities following ABI can also lead to reduced activity levels, and, consequently, reduced participation (Renaud et al., 2018).

This study's primary hypothesis was that Brief BA will increase the mean scores of achievement, closeness and enjoyment of daily activities reported by participants. The study also had four secondary hypotheses: (1) Brief BA will reduce the reported depression symptoms in adolescents with ABI; (2) Brief BA will lead to higher participation levels in adolescents with ABI; (3) Brief BA will lead to better QoL in adolescents with ABI, and; (4) Brief BA will be an acceptable intervention for adolescents with ABI.

## MATERIALS AND METHODS

The study took place during the COVID‐19 pandemic in 2020–2021. The intervention period was during the third national lockdown in the UK, between February and March 2021. COVID‐19 lockdowns caused an increase in depression prevalence and depression symptoms in the UK population of CYP (Shum et al., 2021).

### Participants

#### Recruitment

**FIGURE 1:**
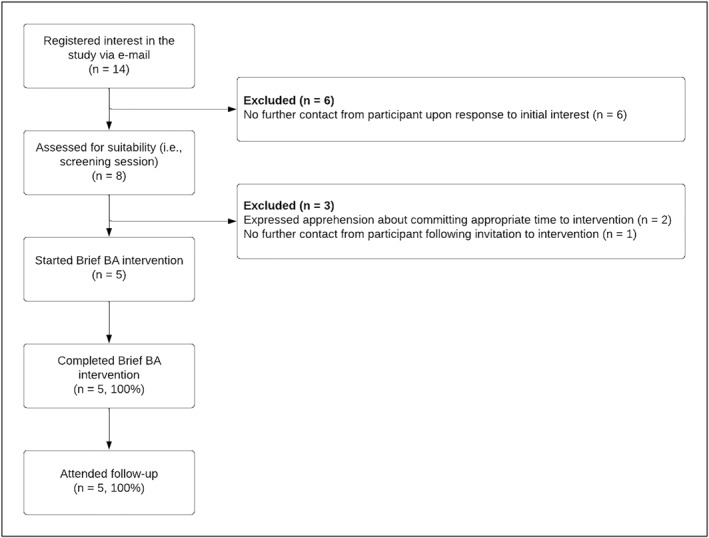
A flowchart representing the study's recruitment, throughput and retention of participants.

Recruitment took place from March 2020 to January 2021. Participants were recruited through the University of Exeter's Child and Adolescent Neuropsychology participant volunteer panel and through various national charities for ABI and neurorehabilitation in CYP. Participant flow through the study is shown in Figure 1.

#### Eligibility criteria

All participants were required to be aged 12–18 years, meet the clinical threshold for symptoms of depression (*T* score = 65+) according to the Revised Children's Anxiety and Depression Scale's (RCADS) Major Depression Disorder Subscale (MDD) for at least one of the child and parent forms (Chorpita et al., 2000), have a history of ABI, and be able to provide informed assent/consent to the study. Those who would not be able to engage in talking therapy, as ascertained at assessment by the main researcher, were excluded.

### Design

**FIGURE 2:**
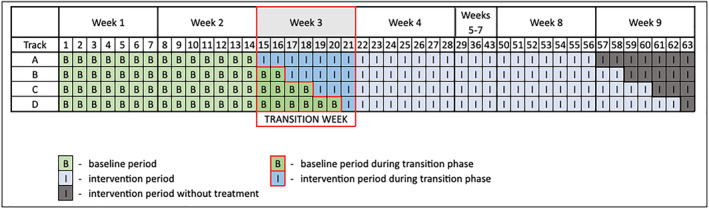
A diagram representing the timeline of events for each track during the data collection period.

A multiple baseline (MBD), single‐case experimental design (SCED) with randomised intervention start points was completed over a 9‐week period, which comprised a minimum 2 weeks of baseline and a minimum 6 weeks of intervention; the transition phase was 1 week long. Each participant was randomly allocated to one of four different tracks, which determined when they started the intervention during the transition phase. Figure 2 represents how the tracks' transition times were staggered, and how this fitted in with the 9‐week data collection period.

### Intervention

#### Brief Behavioural Activation for depression

Brief BA (Pass et al., 2018) comprises eight hourly individual treatment sessions. Parents were invited to be involved for part of sessions 1, 6 and 8. Brief BA is a structured intervention and was delivered according to a treatment protocol, outlined in Appendix S1.

The main researcher (CO) adhered to the ‘Brief BA for Adolescent Depression’ protocol where possible. Due to a need to increase accessibility for a dispersed population, and COVID‐19 restrictions, the intervention was delivered using live online video software. Only three minor adjustments to the protocol were required to make it more amenable for the sample, given the impact of common ABI sequelae, and were decided in advance by the research team; (1) a 5‐minute break in the middle of the session, which participants could opt for if necessary, (2) supplementary phone calls were provided by research interns to support activity recording, and; (3) any information provided was explained at a speed that was comfortable for them and understanding was collaboratively reviewed. No other specific adjustments outside of what would account for typical differences in the adolescent population were needed.

##### Protocol adherence

Treatment adherence checklists, provided by Reynolds and Pass (2021), were used by the clinician (CO) during sessions. Checklists are unique to each session and were ticked off as each checkpoint was reached during the session. Out of the total 40 sessions of Brief BA delivered, full adherence to the checklist was achieved on 37 occasions (92.5% adherence). Any deviance from the checklist was accounted for as a change in the agenda due to what the participant wanted to bring to the session.

### Measures

Primary routine outcome measures (ROMs) were completed daily using Qualtrics. Secondary ROMs were sent via e‐mail to participants, at baseline (T1), immediately post‐treatment (T2) and 4 weeks post‐treatment follow‐up (T3), to be completed and sent back to the researcher.

#### Primary outcome measure

##### Achievement, closeness and enjoyment

Consistent with typical BA procedures, participants completed a daily activity log, where the participant recorded what activities they completed during each day. The participants were asked to rate each activity for its level of ‘achievement’, ‘closeness’ and ‘enjoyment’ out of 10, with ‘0’ being ‘none at all’ and ‘10’ being ‘the highest possible’. Qualtrics, an electronic data collection module, was used to collect these data from participants. A mean daily score for each of the completed ‘achievement’, ‘closeness’, and ‘enjoyment’ ratings was calculated.

#### Secondary outcome measures

##### Depressive symptoms

The RCADS MDD (Chorpita et al., 2000), which includes a child version and a parent version, was used to test Hypothesis 2. The RCADS has high internal consistency (Donnelly et al., 2019), and is highly reliable and valid (Ebesutani et al., 2011). Higher MDD *T*‐scores indicate more severe depression symptoms.

##### Social participation

The Child and Adolescent Scale of Participation (CASP; Bedell, 2004) measures an adolescent with ABI's level of participation at school, home, and community activities (Bedell, 2009). Higher CASP scores indicate higher levels of participation. The child‐report CASP measure was used to test Hypothesis 3.

##### Quality of life

The Paediatric Quality of Life Inventory (PedsQL; Varni et al., 1999) is a measure of QoL in children who are experiencing long‐term health conditions, including neurological conditions. The parent and child core questionnaires were used to test Hypothesis 4. Higher PedsQL scores indicate a better QoL.

##### Study acceptability

This was administered at T3 only. The Treatment Acceptability Questionnaire (TAQ; Hunsley, 1992) consists of six items that are rated on a 7‐point Likert scale and was used to measure treatment acceptability (Hypothesis 5). Acceptability using the TAQ was calculated as a total percentage of the maximum score, with a higher percentage indicating higher study acceptability. The TAQ also allows for collection of qualitative data.

### Procedure

Recruited participants who consented were screened for eligibility over live online video. Those who met eligibility criteria and consented to treatment were offered Brief BA and were invited to an online videocall briefing session with the researcher, which aided participants' understanding of the intervention, how to complete the activity diary and the primary and secondary ROMs on Qualtrics, and session layout.

Participants recorded their activities every day for 2 weeks on the activity diary and entered them onto a similar Qualtrics form. Participants were randomly allocated to an intervention start point across any of the 7 days during week three. Daily activity recordings were collected until the end of week nine. Participants received 6 weeks of Brief BA as per the Brief BA protocol. Four weeks after completing their final session of Brief BA, participants were invited to a follow‐up session. Participants were then debriefed and future considerations for support were discussed.

Secondary ROMs were collected at the first day of the participant's baseline start, the final treatment session, and at the follow‐up session. Data was collected using Qualtrics, for which the link was emailed 24 h before the appointment. The TAQ data was collected at follow‐up.

### Data analysis strategy

#### Hypothesis 1

Visual analysis and statistical analysis using randomisation tests for significance and non‐overlap of pairs for effect size (ES) were performed on the mean achievement, closeness and enjoyment scores (MACES) data, as recommended by Bulté and Onghena (2008). All analyses were performed using the ‘R’ statistical software programme. The functions adhered to for analysis were compiled by Bulté and Onghena (2008, 2009, 2013). For these comparisons, the intervention phase was compared with the baseline phase, analogous to a *t*‐test.

To produce adequate power (>0.80) and detect a large ES, Ferron and Sentovich (2002) recommend collecting at least 20 data points per participant for as few as four participants when employing a MBD in SCED research. To increase the ability to detect a smaller ES, this study was planned to collect 36 data points for up to 10 participants by encouraging participants to provide at least four data points per week. Up to 63 data points were possible due to 9 weeks of daily data collection.

The current study had 57,624 possible randomisation distributions; the author chose to run 1000 randomisation distributions using a Monte Carlo simulation (Bulté & Onghena, 2008; Morley, 2017), as this number is no greater than the possible distributions and higher numbers might not demonstrate superior accuracy despite their increased administrative burden (Heijungs, 2020). Instead of using the typically accepted standard alpha of 0.05 in psychology, the *p‐*value can be compared with an alpha value of 0.1429 for each individual case and for overall group analysis, as this is the lowest possible *p*‐value obtainable with a phase change of 7 days for a one‐tailed test, calculated by dividing 1 by the number of phase change days (i.e., 1/7 = 0.1429; Morley, 2017). A one‐tailed test was chosen given the expected improvement in MACES.

#### Hypotheses 2–4

The Leeds Reliable Change Index (Morley & Dowzer, 2014) was used to measure reliable change in secondary ROMs across all three timepoints. CASP parent data for participation could not be analysed due to an error in storing the data. Effect sizes were calculated using Glass's delta (Δ; Hedges, 1981), using the boundaries suggested by Sawilowsky (2009), outlined in the results.

#### Hypothesis 5

For acceptability ratings, the TAQ provides a descriptive satisfaction level, where the percentage of the highest possible TAQ score was calculated for each participant as a measure of total study acceptability. As the sample size was small, each participant's qualitative response was also recorded.

### Overall intervention effects

Group‐level effect sizes were calculated for all secondary ROMs.

## RESULTS

Five participants completed the 9‐week data collection period; including at least 2 weeks of baseline data. All participants had missing data points; two participants (1 and 3) were excluded from the primary analysis of Hypothesis 1 due to having over 50% of missing data. Of the three participants achieving over 50% of data, Participant 5 had three missing data points whilst the other two (Participants 2 and 4) had 24 missing data points each out of the total maximum of 63 data points (seven data points per week for 9 weeks).

The primary reasons for missing data as reported by participants during sessions were forgetting, fatigue, and tedium. For visual analysis and non‐overlap of pairs calculations (randomisation tests could be calculated with missing data) of the MACES ratings, any missing data were retrospectively managed using median substitution, where the median for each intervention phase was calculated and put in place of missing data dependent on the phase. All participants who completed the intervention were still put forward for analysis of secondary measures (Hypotheses 2–5), as data from these outcomes following Brief BA were still of interest.

All participants' parents attended the sessions 1, 6 and 8, to which they were invited. All parents reported supporting their child with the between‐session work and outcome measures between each session; though, the frequency and duration was not recorded.

### Participants

Table 1 outlines participant characteristics, including brief information about their ABI.

**TABLE 1:** A summary of each participant's demographic characteristics at the time of screening.

Ppt. no.	Age^a^ (years)	Gender	Ethnicity^b^	IMD decile^c^	Lives with	Screening RCADS MDD *T*‐score	Type of ABI^d^	ABI severity^d^	Time since ABI (years)^a^	Notable impairments^d^
1	14	F	‘White European’	9	Mother	C = 89^f^ P = 78^f^	General encephalomyelitis leading to a coma; congenital brain injury under investigation	Participant unsure	10	Conceptual reasoning Fatigue Participation Physical difficulties Processing speed Visual problems
2	15	F	‘White British’	9^e^	Mother Father Sister Sister	C = 92^f^ P = 93^f^	8 cm subdural abscess pressing on right frontal lobe	Participant unsure	2.5	Attention Emotional regulation Fatigue Impulsivity Initiation Keeping routine Memory Noise sensitivity Processing speed
3	14	M	‘British Asian’	3	Mother Father Brother Brother	C = 76^f^ P = 99^f^	Tumour on posterior fossa on two separate occasions; surgery, radiotherapy and chemotherapy on both occasions	‘Severe’	1st: 7 2nd: 2	Cognitive inflexibility Flat affect Keeping routine Noise sensitivity Processing speed Short‐term memory Visual problems Balance difficulties
4	15	F	‘White British’	9	Mother	C = 59 P = 87^f^	6 cm atypical teratoid tumour on left frontal and temporal lobes; multiple surgeries, radiotherapy and chemotherapy	‘Mild’	4	Appetite Attention Fatigue Noise sensitivity Processing speed Short‐term memory Sleep
5	16	F	‘Mixed White and Black Caribbean’	10	Mother	C = 58 P = 82^f^	Traumatic brain injury and contrecoup; hit head‐on by heavy object	Participant unsure	15	Contextual reasoning Fatigue Flat affect Literal thinking Noise sensitivity Processing speed Short‐term memory

Abbreviations: ABI, acquired brain injury; C, child version; F, female; IMD, Index of Multiple Deprivation; M, male; P, parent version; Ppt. no., participant number; RCADS MDD, Revised Child Anxiety and Depression Scale Depression Subscale.

^a^ Values accurate at the start of the intervention.

^b^ Ethnicity as reported by the participant with the support of their parent.

^c^ IMD (Ministry of Housing, Communities & Local Government, 2019) overall decile at home postcode; socioeconomic status.

^d^ All data as reported by participants and their parents at screening, based on experience/reported information given by professionals.

^e^ Postcode in Scotland; Scottish IMD (Scottish Government, 2021) was used.

^f^ Clinically significant score according to Chorpita et al. (2000).

### Hypothesis 1

#### Visual analysis

**FIGURE 3:**
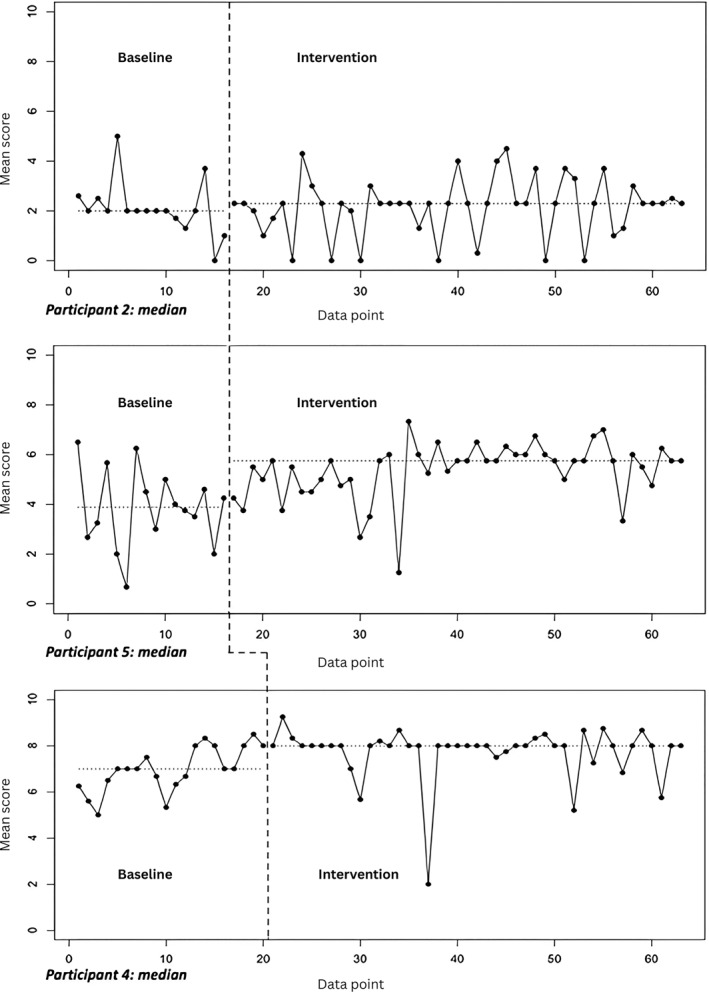
The median of each participant's daily mean ‘achievement’ scores for each phase.

**FIGURE 4:**
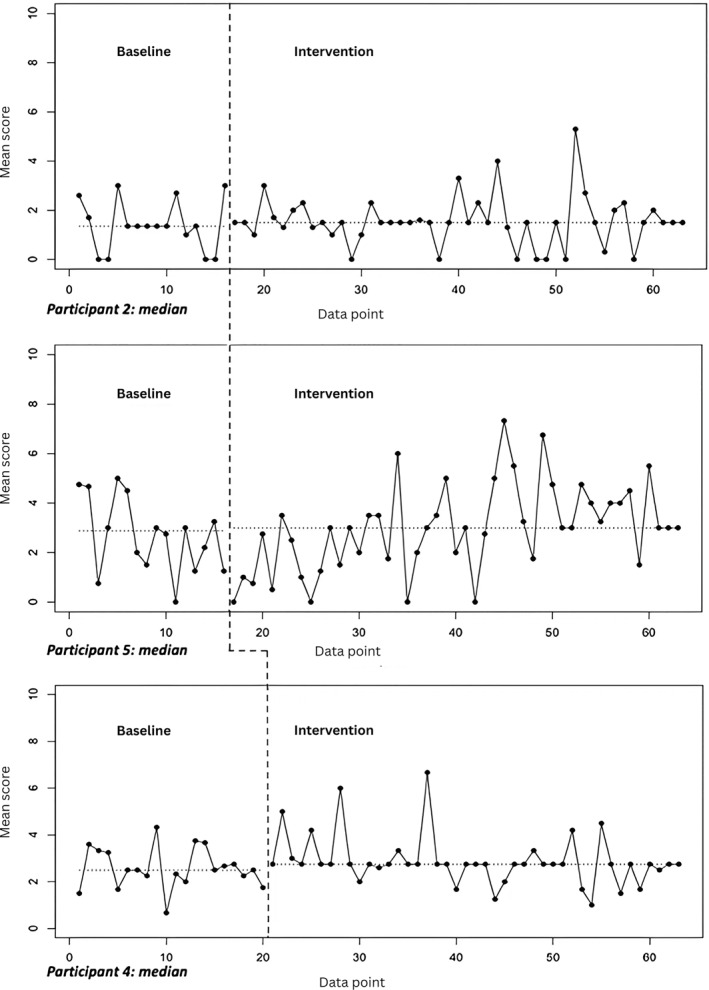
The median of each participant's daily mean ‘closeness’ scores for each phase.

**FIGURE 5:**
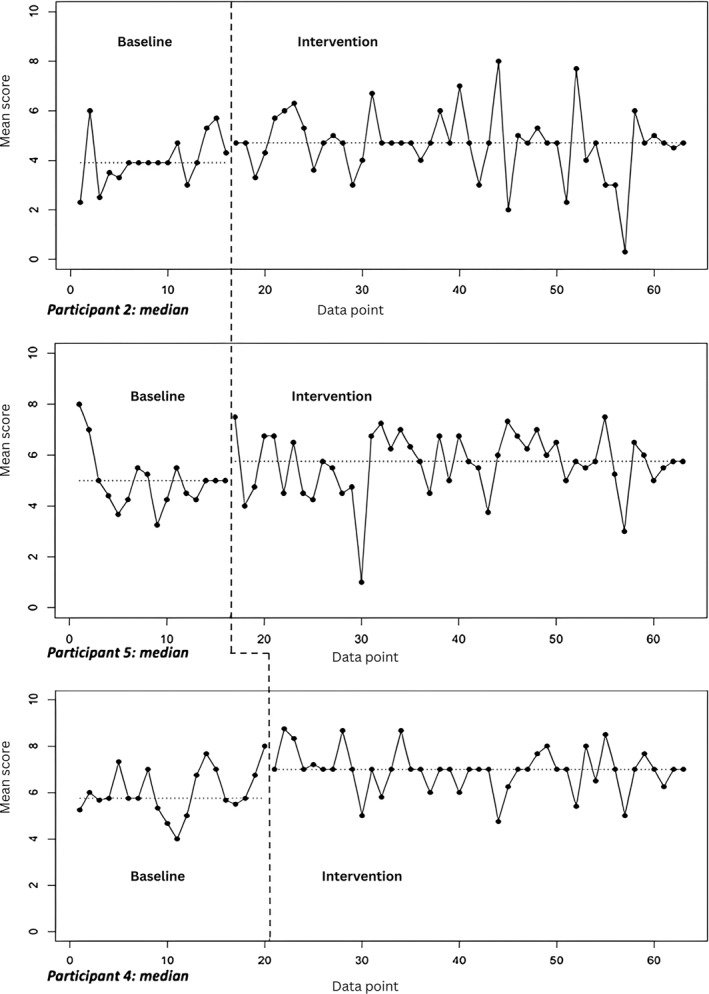
The median of each participant's daily mean ‘enjoyment’ scores for each phase.

**FIGURE 6:**
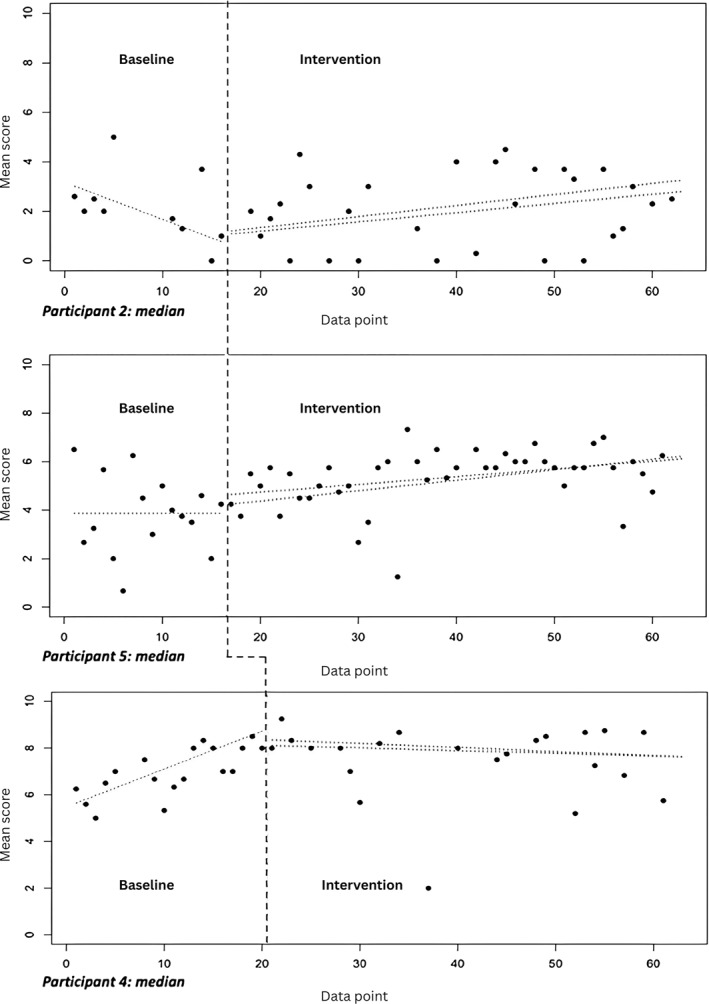
The trend of each participant's daily mean ‘achievement’ scores for each phase.

**FIGURE 7:**
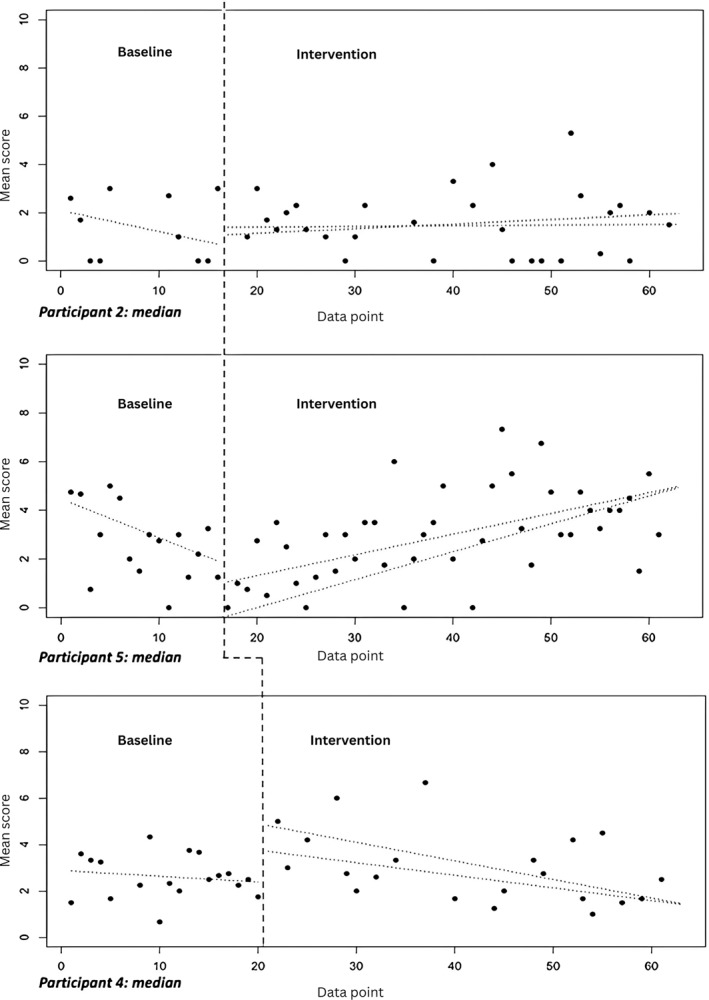
The trend of each participant's daily mean ‘closeness’ scores for each phase.

**FIGURE 8:**
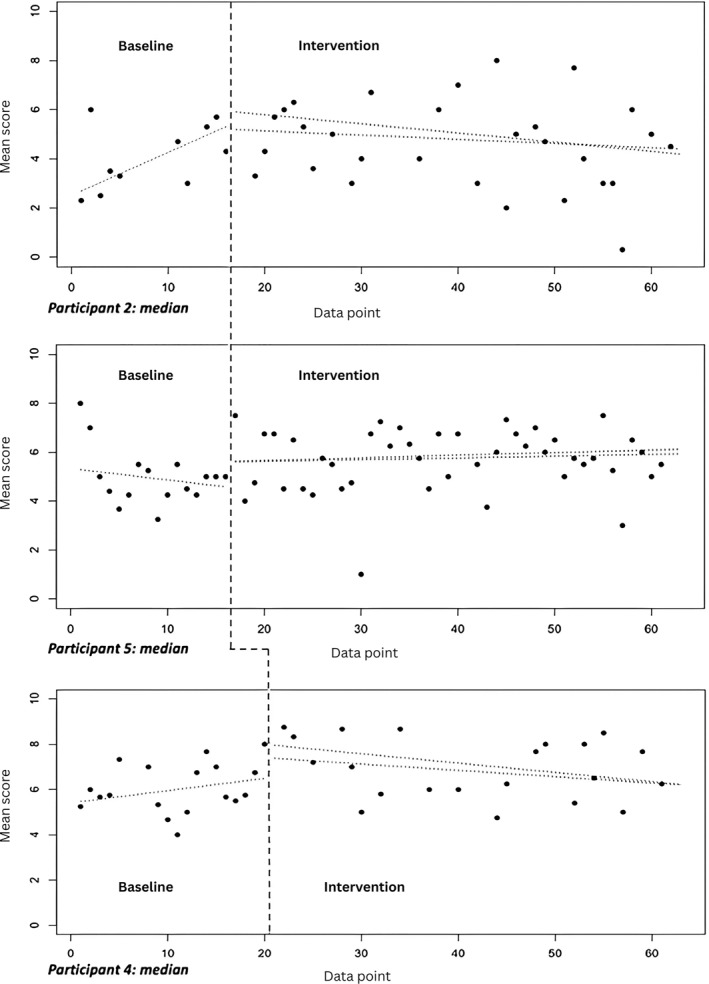
The trend of each participant's daily mean ‘enjoyment’ scores for each phase.

The MACES data for visual analysis of central tendency are displayed in Figures 3, 4, 5 for Participants 2, 4 and 5, separated by the three target areas: achievement, closeness and enjoyment. The corresponding data for visual analysis of trends are displayed in Figures 6, 7, 8.

##### Participant 2

Compared to baseline, Participant 2 showed a very slight increase in median ‘achievement’ and ‘enjoyment’ scores, with very little change in their median ‘closeness’ scores during the intervention. Participant 2 reported predominantly low ‘achievement’ scores, particularly in the intervention phase. Trend analysis showed gradually increasing ‘achievement’ and very slightly increasing ‘closeness’ scores over time during the intervention phase. Though, ‘enjoyment’ scores were slightly decreasing, and showed more extreme values during the intervention phase.

##### Participant 5

Compared to baseline, Participant 5 showed a marked increase in median ‘achievement’ scores and a small increase in ‘enjoyment’ scores, with very little change in their median ‘closeness’ scores during the intervention. Participant 5 showed little daily fluctuation in ‘enjoyment’ scores during the intervention period and attributed this to ‘good mood’. Participant 5's trend data showed slightly increasing ‘enjoyment’ scores and markedly increasing ‘achievement’ and ‘closeness’ scores during the intervention phase.

##### Participant 4

Participant 4 started the intervention phase 4 days after Participants 2 and 5, and showed the most stable scores overall. Compared to baseline, Participant 4 showed an increase in median ‘achievement’ and ‘enjoyment’ scores, with very little visible change in her median ‘closeness’ scores during the intervention. Participant 4's trend data demonstrated a slightly decreasing ‘achievement’ scores and markedly decreasing ‘closeness’ and ‘enjoyment’ scores over time during the intervention phase; all of which started at a high score.

#### Randomisation and effect size tests

Table 2 shows the mean scores, non‐overlap of pairs effect sizes, and significance of each participant's mean scores for each phase for ‘achievement’, ‘closeness’ and ‘enjoyment’.

**TABLE 2:** Mean ‘achievement’, ‘closeness’ and ‘enjoyment’ scores, effect sizes and randomisation tests for each participant and the overall totals, means and figures across participants.

Ppt.	Phase duration (days)		Mean score (SD)		NAP^a^	*p*‐value	Sig.?^b^
	Baseline	Intervention	Baseline	Intervention			
Achievement							
2	16	47	2.18 (1.40)	2.08 (1.50)	0.606	0.946	No
4	20	43	6.98 (1.04)	7.47 (1.62)	0.745	0.275	No
5	16	47	3.85 (1.59)	5.34 (1.18)	0.787	0.192	No
Overall	52	137	4.75 (2.38)	4.84 (2.45)	0.714	0.719	No
Closeness							
2	16	47	1.40 (1.35)	1.57 (1.32)	0.583	0.262	No
4	20	43	2.60 (0.93)	3.03 (1.56)	0.615	0.052	Yes
5	16	47	2.68 (1.52)	2.97 (1.83)	0.551	0.938	No
Overall	52	137	2.36 (1.34)	2.55 (1.74)	0.584	0.419	No
Enjoyment							
2	16	47	4.06 (1.33)	4.62 (1.77)	0.692	0.2	No
4	20	43	6.06 (1.08)	6.92 (1.34)	0.755	0.214	No
5	16	47	4.99 (1.17)	5.66 (1.30)	0.711	0.095	Yes
Overall	52	137	5.22 (1.40)	5.62 (1.67)	0.721	0.858	No

Abbreviations: NAP, non‐overlap of all pairs; Ppt., participant; SD, standard deviation.

^a^ NAP ranges: 0–0.65 = ‘weak’; 0.66–0.92 = ‘medium’; 0.93–1 = ‘large’ (Parker & Vannest, 2009).

^b^ As compared to allocated 0.1429 significance alpha value.

##### Overall results

Findings from non‐overlap of pairs analysis showed that, compared to baseline, the overall ES for ‘achievement’ was in the ‘medium’ range, ‘closeness’ was in the ‘small’ range and ‘enjoyment’ was in the ‘medium’ range. Compared to baseline, randomisation tests showed no statistically significant overall changes in the intervention phase.

##### Individual results

Compared to the baseline phase, Participant 4 showed a significant (randomisation tests) but small (non‐overlap of pairs) change in ‘closeness’ scores, and Participant 5 showed a significant, medium change in ‘enjoyment’ scores during the intervention phase. Medium changes in ‘achievement’ scores were noted for Participants 4 and 5 during the intervention phase compared to baseline but these were not statistically significant.

### Hypotheses 2–4

#### Individual results

Descriptive statistics for all secondary measures for all participants are shown in Table 3. Reliable change and clinically significant change at timepoints compared to baseline are also reported. Compared to baseline, Participants 1, 3 and 4 reported clinically significant change in MDD child scores at post‐treatment, and whilst Participants 1 and 4 maintained clinically significant change at follow‐up, all participants reported reliable change by follow‐up. In parents' RCADS depression subscale scores, compared to baseline, Participants 1, 4 and 5 showed clinically significant change and all participants showed reliable change by follow‐up. Compared to baseline, Participants 2 and 3 showed reliable change in child PedsQL scores at least at one time‐point, whilst all participants' parent PedsQL scores showed reliable change by follow‐up. No other statistically significant changes were noted.

**TABLE 3:** Secondary routine outcome measure (ROM) scores for each participant at baseline, post‐treatment and follow‐up, and whether differences indicate reliable change and clinically significant change.

	Timepoint		
Routine outcome measure	Baseline (T1)	Post‐treatment (T2)	Follow‐up (T3)
RCADS MDD child raw score (*T*‐score)^a^			
Participant 1	17 (75)	10^d^ (56)^e^	7^d^ (48)^e^
Participant 2	23 (92)	19 (81)	15^d^ (70)
Participant 3	19 (84)	6^d^ (48)^e^	14^d^ (70)
Participant 4	13 (65)	10 (56)^e^	7^d^ (48)^e^
Participant 5	12 (56)	3^d^ (36)	1^d^ (31)
RCADS MDD parent raw score (*T*‐score)^a^			
Participant 1	14 (81)	11^d^ (72)	7^d^ (59)^e^
Participant 2	18 (93)	14^d^ (81)	16^d^ (87)
Participant 3	16 (89)	13^d^ (80)	11^d^ (73)
Participant 4	16 (87)	8^d^ (62)^e^	8^d^ (62)^e^
Participant 5	13 (76)	9^d^ (63)^e^	7^d^ (57)^e^
PedsQL child^b^			
Participant 1	40.2	47.8	47.8
Participant 2	32.6	‐	43.5^d^
Participant 3	44.6	55.4^d^	42.4
Participant 4	61.7	55.4	65.2
Participant 5	53.3	50.0	50.0
PedsQL parent^b^			
Participant 1	21.7	‐	40.2^d^
Participant 2	38.0	43.5	48.9^d^
Participant 3	42.4	62.0^d^	58.7^d^
Participant 4	44.6	65.2^d^	66.3^d^
Participant 5	28.3	51.1^d^	47.8^d^
CASP child^c^			
Participant 1	‐	67.1	76.3
Participant 2	68.8	‐	77.5
Participant 3	82.9	73.8	92.1
Participant 4	85.0	88.2	88.2
Participant 5	53.8	57.5	53.8

*Note*: CASP parent data could not be included due to an error in storing the data. ‐, Missing data.

Abbreviation: CASP, Child and Adolescent Scale of Participation.

^a^ Raw RCADS score as described in Chorpita et al. (2015).

^b^ Mean total scores as described by Varni et al. (1999).

^c^ Total raw score as described by Bedell (2004).

^d^ Reliable change since baseline.

^e^ Clinically significant change from ‘caseness’ to ‘recovery’.

#### Overall results

Effect sizes for overall scores were calculated for each secondary ROM using Glass's delta (Δ; Hedges, 1981) and are shown in Table 4 for baseline to post‐treatment and Table 5 for baseline to follow‐up. Effect sizes for RCADS depression subscale child scores at post‐treatment and follow‐up compared to baseline, RCADS depression subscale parent scores at post‐treatment and follow‐up compared to baseline, and PedsQL parent scores at post‐treatment and follow‐up compared to baseline were ‘very large’. Effect sizes for PedsQL child scores at post‐treatment and follow‐up compared to baseline, and CASP child scores at follow‐up compared to baseline were small. The ES for CASP child scores at post‐treatment compared to baseline was very small.

**TABLE 4:** Mean routine outcome measure (ROM) scores and standard deviations for all participants at baseline (T1) and post‐treatment (T2), with calculated effect sizes using glass's delta.

Routine outcome measure	Timepoint mean scores (SD)		ES (Δ)^a^
	T1	T2	
RCADS MDD child (*n* = 5)	16.8 (4.49)	9.6 (6.02)	1.60
RCADS MDD parent (*n* = 5)	15.4 (1.95)	11.0 (2.55)	2.26
PedsQL child (*n* = 4)	49.95 (9.54)	52.15 (3.86)	0.23
PedsQL parent (*n* = 4)	38.33 (7.22)	55.45 (9.99)	2.37
CASP child (*n* = 3)	73.90 (17.44)	73.17 (15.36)	−0.04

Abbreviation: ES, effect size.

^a^ Δ ranges: 0.01–0.19 = ‘very small’; 0.20–0.49 = ‘small’; 0.50–0.79 = ‘medium’; 0.80–1.19 = ‘large’; 1.20–1.99 = ‘very large’; 2.0+ = ‘huge’ (Sawilowsky, 2009).

**TABLE 5:** Mean routine outcome measure (ROM) scores and standard deviations for all participants at baseline (T1) and follow‐up (T3), with calculated effect sizes using glass's delta.

Routine outcome measure	Timepoint mean scores (SD)		ES (Δ)^a^
	T1	T3	
RCADS MDD child (*n* = 5)	16.8 (4.49)	8.8 (5.76)	1.78
RCADS MDD parent (*n* = 5)	15.4 (1.95)	9.8 (3.83)	2.87
PedsQL child (*n* = 5)	46.48 (11.33)	49.78 (9.16)	0.29
PedsQL parent (*n* = 5)	35.00 (9.72)	52.38 (10.19)	1.79
CASP child (*n* = 4)	72.63 (14.47)	77.90 (17.21)	0.36

Abbreviations: CASP, Child and Adolescent Scale of Participation; ES, effect size using Glass's delta (Δ; Hedges, 1981).

^a^ Δ ranges: 0.01–0.19 = ‘very small’; 0.20–0.49 = ‘small’; 0.50–0.79 = ‘medium’; 0.80–1.19 = ‘large’; 1.20–1.99 = ‘very large’; 2.0+ = ‘huge’ (Sawilowsky, 2009).

### Hypothesis 5

#### Quantitative findings

The mean TAQ rating for Brief BA given by participants was 36.6 (SD 3.07), which was 87% of the maximum score, ranging from 76% to 95%. Participants scored highest for its ethicality and low possibility of negative side effects (91%) and lowest for intervention acceptability (80%). Participants scored 86% for psychologist knowledge and for the potential wider effectiveness of Brief BA, and 89% for trust in the psychologist.

#### Qualitative findings

Table 6 lists the qualitative feedback given by participants in the TAQ, separated by answers to questions about what participants ‘liked’ and ‘did not like/improvement suggestions’.

**TABLE 6:** Feedback from each participant, grouped as ‘likes’, and ‘did not likes/improvement suggestions’.

Ppt. no.	Likes	Did not likes/improvement suggestions
1	‘Everything was really well explained and I got to talk to someone who was also a young person about my issues’	‘I think it could have gone on longer’
2	‘I learned how to tell normal teenage feelings to post brain injury feelings’	NA
3	‘The psychologist made sure I was comfortable speaking’	‘I wouldn't want it to be online’
4	‘I found it helpful to just go over everything I did and valued in the day’	NA
5	‘[It was] interesting and made me think about how my mood can affect others’	NA

Abbreviations: NA, did not answer; Ppt. no., participant number.

## DISCUSSION

The current study aimed to investigate the efficacy of Brief BA for improving MACES scores, and consequently, depression, in adolescents with ABI. All measures were likely to have been influenced by social restrictions put in place by the UK Government in response to the COVID‐19 pandemic; participation (Hypothesis 4), as a measure driven by social interaction, and closeness (part of Hypothesis 1) were most likely affected, particularly as some questions in the CASP ask about ‘connectedness’ and activities focusing on ‘closeness’ would have mainly involved spending time with others.

Despite an overall lack of significance for Hypothesis 1, results suggested that some participants might have found some activity types more attractive than others. Notably, ‘enjoyment’ scores were considerably higher for Participants 2 and 5 across both phases when compared with ‘achievement’ and ‘closeness’, which might suggest that enjoyment‐oriented activities are likely to be more attractive than achievement or closeness‐orientated activities.

In the visual trend analysis, Participant 2 showed increasing MACES scores in ‘achievement’ and ‘closeness’. Participant 5 showed increasing MACES scores in ‘achievement’, ‘closeness’ and ‘enjoyment’. This suggests that as the intervention was gaining momentum, Participants 2 and 5 were performing activities that gave them more achievement, closeness and enjoyment. Had the intervention gone on for longer with more sessions, this might have resulted in increased MACES and possibly RCADS depression subscale, PedsQL and CASP gains, if MACES were the mechanism of change in Brief BA. Participant 5's higher MACES data input (60/63 timepoints) might have given a more reliable picture of how MACES were affected during the intervention phase, relative to the other participants' data.

In contrast, Participant 4 showed decreasing scores for all three activity types during the intervention phase, despite a large immediate difference in mean MACES scores at the start of the intervention phase compared to baseline. Participant 4 reported regularly experiencing difficulties with fatigue more than other participants, which might have resulted in difficulties maintaining the activities with higher MACES that they immediately implemented at the beginning of the intervention. Perhaps an introduction of protected breaks in Participant 4's schedule to adjust for this might have mitigated a decrease in scores over time; Wheatcroft and Malley (2020) suggest that taking regular breaks is an important part of ‘pacing’ when managing such fatigue, leading to improved outcomes when managing activity levels.

Activity logs are a key component of Brief BA, and the extent of missing data for Participants 1 and 3 (>60%) demonstrates how difficult some adolescents with ABI might find completing these tasks. Participant 3 gave continuously low scores across all activity types, whilst Participant 1 gave either very low or very high scores to activities. This might allude to difficulties with activity appraisal due to apathy (Tate et al., 2020), limited insight (Headway, 2016), and proneness to inflexible thinking (Whiting et al., 2017), which are common in people with ABI.

When seen for their follow‐up session, most participants suggested to the therapist that their ABI might have impacted their ability to keep a routine and track their daily activities, mainly due to difficulties with self‐regulation and short‐term memory; aligning with findings from Cantor et al. (2008) and Hart and Evans (2006). All participants and their parents also commented on how different their outcomes might have been had it not been for COVID‐19 restrictions. For example, Participant 3 was unable to do his favourite activities: shopping and seeing his cousins. These issues may have impacted ROMs results, making them potentially less reliable. Future research could repeat the current study in a post‐COVID era to investigate this.

Hypothesis 2 predicted a reduction in the reported symptoms of depression in participants following Brief BA. The hypothesis was mostly supported by the MDD results. Participants 1 and 3 both showed clinically significant change at post‐intervention compared to baseline in RCADS depression subscale scores; yet Participant 3 could not maintain this improvement after the intervention had finished, which might indicate having the space to reflect on activities and mood with a therapist is helpful but difficult when done independently, especially when MACES scores are low. This may be accounted for by non‐specific factors of therapy driving mood improvements.

In terms of possible mechanisms for change in depression scores, the evidence supporting the influence of activity scores on depression in this study is inconclusive. Only the data from Participants 2, 4 and 5 could undergo visual and randomisation test analysis for significant changes in MACES and there was a lack of significant change in MACES at post‐treatment compared to baseline. Nevertheless, the mechanisms of increasing positive reinforcement, reducing negative reinforcement, and increased awareness of activities and their impact on mood (Reynolds & Pass, 2021) might well have had a significant impact on outcomes, as these were the main focus in sessions. When the previously discussed difficulties with insight in adolescents with ABI are considered, perhaps MACES might not fully reflect increases in positive reinforcement and reductions in negative reinforcement. It could also be argued that participants benefited from having the space to discuss with a therapist how their mood and activity scores are linked. Watson et al. (2021) found that young people reported connecting with values and self‐monitoring as playing an important role in managing anhedonia. This is further supported by participants' feedback in Table 6 of the current study.

Hypotheses 3 and 4 predicted an improvement in the participation and QoL of participants following Brief BA. Compared to baseline, participation scores showed no change for all participants and child QoL scores were variable at all timepoints; however, it is notable that parents' QoL scores showed reliable change for all participants and a very large ES. Future research could investigate what the adolescents themselves value as determinants of QoL compared with their parents. There may have also been an element of parental bias, whereby they may have wanted the intervention to be successful.

This is the first study to investigate the efficacy of Brief BA in increasing activity scores in adolescents with ABI. The SCED methodology was the most appropriate method, as it provides a robust insight into how the intervention can be applied in clinical settings for less common presentations (Morley, 2017). This is particularly appropriate given the availability of Brief BA across England and its cost‐effectiveness (Pass et al., 2018; Richards et al., 2016). The study adhered closely to the protocol that would typically be delivered in NHS services, meaning the study allows for a close exploration of how adolescents with ABI respond to standard care for adolescents with depression. Future research could build on the current study by similarly investigating Brief BA for a wider range of neurological conditions, or perhaps consider a more powerful, controlled trial with a larger number of adolescent participants with depression following ABI. Now that COVID‐19 restrictions are lifted, a repeat of this study might produce different results, as adolescents will be able to continue with their usual activities.

Another strength was the study's use of live online video, which meant the intervention could be rolled out to a population that typically does not have access to many services at all. This meant the current study could be delivered from a small hamlet in mid‐Devon to as far as south‐west Scotland. The successful delivery and acceptability of this intervention adds to current research into the feasibility of online neuropsychology service delivery (Bennett et al., 2021); future studies or established therapy providers could consider live online delivery of interventions for adolescents with ABI, thus increasing access to evidence‐based treatments. This suggests that serious consideration should be given to the potential for such interventions to be rolled out nationally or even internationally, for adolescents with ABI or for adolescents as a whole population.

Though, considerations must be made to ensure inclusivity for those from lower socioeconomic backgrounds too. The use of the Internet and online intervention delivery may disadvantage many adolescents from lower socioeconomic backgrounds, who are much less likely to have access to the Internet or the required software compared with peers from higher socioeconomic backgrounds (Local Government Association, 2021). This reflects a limitation of the current study.

The current study has some further limitations. Firstly, MACES ROMs had a lot of missing data. Whilst activity monitoring is a key part of Brief BA, its use as an outcome measure may have been burdensome for participants. In sessions, participants sometimes reported filling in fewer activities to reduce the administrative burden or did not fill it in due to fatigue, forgetfulness or tedium; despite this, participants did not provide this as negative feedback in the Treatment Acceptability Questionnaire. All participants tended to report the minimum three activities required per day, potentially due to the administrative burden, which meant it was not possible to see whether the frequency of activity had increased after the intervention. The daily monitoring of activities may also have resulted in fewer study participants, as many might have been put‐off by the amount of data entry required. It would have been difficult to investigate changes in MACES without recording daily activities.

Future studies may wish to mitigate forgetfulness and fatigue by considering the embedment of automatic daily reminders, increased contact with research interns through more regular supplementary phone calls, or encouraging specific rewards, particularly from parents, to encourage more frequent data inputting. As activity monitoring is a key concept of Brief BA, these initiatives might have resulted in increased primary and secondary gains for participants, and are worth considering when implementing Brief BA for this population clinically.

The current research presents several clinical implications. CYP with ABI should be able to access mainstream low‐intensity interventions such as Brief BA with minor adjustments recommended by the authors, such as more sessions, frequent check‐in phone calls, and brief mid‐session breaks. As discussed, the findings suggest that having the space to reflect on and discuss emotions, mood and how they impact on activity scores may be a potentially useful intervention in itself (Table 6; Watson et al., 2021). More research is needed before drawing more definitive conclusions. For now, services and charities for paediatric ABI might consider trialling Brief BA in their own services, with robust service evaluation to explore its efficacy, acceptability and feasibility in their specific settings. As the prevalence of depression in CYP with ABI is high (Hendry et al., 2020; Schachar et al., 2015) and Brief BA is a relatively cost‐effective therapy compared to most other therapies, this might be a worthwhile investment for ABI services and charities. The demonstrated improvements in QoL as reported by participants' parents might also mean Brief BA could be useful even if depression is not necessarily the target problem, as it mainly focuses on emotional and behavioural regulation, and valued activities.

## CONCLUSION

The current study has provided, for the first time, support for Brief BA as a suitable and acceptable intervention for adolescents with depression following ABI. MACES findings were mixed, with significant individual improvements in either ‘closeness’ or ‘enjoyment’ for three participants who were suitable for analysis. However, the overall study findings suggest that focusing on valued activities, increasing positive reinforcement and reducing negative reinforcement, and how these mechanisms link to mood had a positive effect on every participant's depression scores and parent‐reported QoL, providing supporting evidence of efficacy. Given the likely impact of the COVID‐19 pandemic on the outcomes of this study, these results are promising and should be investigated further in research and evaluation of clinical practice.

## AUTHOR CONTRIBUTIONS

**Conor R. O'Brien:** Conceptualization; data curation; formal analysis; investigation; methodology; writing—original draft; writing—review and editing. **Jenny Limond:** Conceptualization; data curation; formal analysis; methodology; supervision; writing—review and editing. **Shirley Reynolds:** Conceptualization; methodology; writing—review and editing. **Laura Pass:** Conceptualization; methodology; writing—review and editing. **Anna‐Lynne R.Adlam:** Conceptualization; data curation; formal analysis; methodology; supervision; writing—review and editing.

## CONFLICT OF INTEREST STATEMENT

The authors declare no conflicts of interest.

## ETHICAL CONSIDERATIONS

Ethical approval was provided by the University of Exeter Psychology ethics committee. All participants were asked for their assent/consent at the screening phase and the intervention phase. Participants were provided with a clear, age‐appropriate participant information sheet before signing consent forms. Consent forms provided clear statements, which required participants' initials to show that each statement had been fully understood. Parents were also provided with a participant information sheet. Participants under 16 were able to provide assent and their parents/caregivers were required to provide consent in order to take part. Participants over 16 did not require parental consent and provided their own consent. All participants were informed of their right to withdraw without penalty.

## Supporting information

Supporting Infromation S1

## Data Availability

Data is stored securely as per the University of Exeter's Data Protection policy. Data can be provided upon request by e‐mailing the corresponding author.
