# Supplementary material for: Evaluating Brief Behavioural Activation for depression in adolescents with acquired brain injury using a single‐case experimental design
Source: JCPP Adv. 2025 Aug 13;6(2):e70030. doi: 10.1002/jcv2.70030 (PMC13260684; doi:10.1002/jcv2.70030)
Supplement: Supplementary file 1 — Supporting Infromation S1 [file JCV2-6-e70030-s001.docx]

**Appendix S1**

*Structured Session Overview of Brief BA for Adolescent Depression, Taken from Pass, Lejuez, & Reynolds (2018)*

| Session | Young person content | Parent content | Homework |
| --- | --- | --- | --- |
| 1 | Introduction to Brief BA approach and rationale, session workbook. | Attend part of session (rationale, structure of Brief BA), parent workbook. | Activity log. |
| 2 | Review of Brief BA approach, review of activity log, session workbook. | Parent workbook. | Activity log. |
| 3 | Review of activity log, introduction to values, session workbook. | Parent workbook. | Activity log, values. |
| 4-5 | Review of values, plan valued activities across life areas, session workbook. | Parent workbook. | Valued activities. |
| 6 | Review of progress, introduction to problem-solving and contracting, session workbook. | Attend part of session (review, problem-solving, contracts), parent workbook. | Valued activities. |
| 7 | Review of progress, identification of activities to continue working towards, session workbook. | Parent workbook. | Valued activities. |
| 8 | Review of progress, relapse prevention, session workbook, relapse prevention handout. | Attend part of session (review, relapse prevention), parent workbook. |  |
| Review | Review of progress, plan for further input/discharge. | Attend part of review. |  |
